# Vaccine management practices among healthcare workers in Morogoro, Tanzania: a cross-sectional study

**DOI:** 10.1186/s40545-022-00496-y

**Published:** 2022-11-30

**Authors:** Bonaventura Nestory, Mackfallen Anasel, Jean Baptiste Nyandwi, Domina Asingizwe

**Affiliations:** 1 EAC Regional Centre of Excellence for Vaccines, Immunization, and Health Supply Chain Management, College of Medicine and Health Sciences, University of Rwanda, Kigali, Rwanda; 2 Ministry of Health, Immunization and Vaccine Development Program, Dodoma, Tanzania; 3 School of Public Administration and Management, Mzumbe University, Mzumbe Morogoro, Tanzania

**Keywords:** Temperature, Vaccine, Cold chain management, Tanzania

## Abstract

**Background:**

Effective vaccine management is essential to maintain the quality of vaccines, minimise wastages, and prevent missed opportunities for vaccination at service delivery points.

**Objectives:**

This study aims to assess vaccine management practices among vaccinators at health facilities in the Morogoro region, Tanzania.

**Methodology:**

A descriptive cross-sectional study design involved health workers from 77 health facilities offering vaccination services. The study population consisted of vaccine handlers and vaccinators working in public health facilities in the Morogoro region. The vaccine management practices were assessed using data collected from ledgers and the Vaccine Information Management System (VIMS). The temperature records were downloaded from the Fridge-tag® 2 and Coldtrace5 devices.

**Results:**

The findings indicated that 65 (84%) health facilities had functional refrigerators and are using power from 26 (34%), 28 (36%), and 23 (30%) of grid electricity, solar, and Liquefied Petroleum Gas (LPG), respectively. Besides, 27 (35%) health facilities have an alternative energy source as a backup. In general, healthcare workers had a good knowledge of cold chain management, including the World Health Organization recommended storage temperatures for vaccines. Furthermore, vaccine stockout was found in 12 (15.6%) health facilities for at least one antigen and 4 (5.1%) health facilities for all five antigens under observation. This current study also revealed that the average calculated vaccine wastage rates for DTP, Measles–Rubella and Rotavirus vaccines were 7%, 19%, and 15%, respectively. More than half of health workers did not perform monthly temperature data reviews. In addition, poor performance led to high wastage rates, including the Rotavirus vaccines, and a change in VVM to discard points. Finally, a small number of 5 (6.5%) health facilities consecutively reported temperature exposure beyond + 8 Celsius (between 5.9 and 281 h).

**Conclusions:**

Healthcare workers’ vaccine and cold chain management knowledge were good for temperature data reading and documentation. However, the practices were poor for some health facilities. The gaps observed in this study inform health managers and policymakers toward establishing interventions to improve health workers' knowledge and practice, including mentorships, supervision, and training to guarantee that each child in all communities reaps the benefits of immunisation services.

## Background

Vaccines are immunobiological perishable products used to prevent diseases by activating antibodies in human bodies [1]. Vaccines are used to prevent and control more than 20 life-threatening infectious diseases, including paralysis, measles, and meningitis. The World Health Organization (WHO) report of 2021 indicated that only 83% of infants were vaccinated with three doses of diphtheria–tetanus–pertussis (DTP3)-containing vaccines in 2020, showing a 3% drop from 86% vaccination coverage for the same antigen that was reported in 2019 [2].

Vaccines' potency is affected by exposure to both high temperatures and freezing temperatures [3]. The freezing can cause changes in physical appearance and lead to loss of potency of diphtheria and tetanus vaccines due to the damage of the adjuvant gel structure of toxoids. Furthermore, Oral poliomyelitis is identified as the least stable vaccine unless it is stored at low temperatures to maintain its potency. The exposure of vaccines to suboptimal temperatures is a widespread problem observed at all levels of the healthcare systems in both developing and developed countries causing vaccine damage or delivery of sub-potent vaccine [4–6].

The WHO recommends that storage of routine childhood vaccines at higher stores and health facilities be optimised to avoid vaccine damage by exposure to heat, freezing, and light [7]. It is recommended that vaccines should be stored between + 2 to + 8 °C, except Oral Polio vaccines (OPV), so as to preserve their quality, efficacy, and safety [8–10]. Exposure of vaccines to sub-zero temperatures (< 0° Celsius) during storage at health facilities can equally affect the potency of heat-sensitive vaccines, such as Tetanus Containing vaccines (TCV), diphtheria–tetanus–pertussis containing vaccines (DTP), Pneumococcal Conjugate vaccines (PCV) and Hepatitis B which are used in many nations, including Tanzania [11]. Moreover, exposed vaccines can no longer protect recipients against targeted diseases when affected by extreme temperatures [12]. Therefore, health workers must maintain the cold chain system to preserve vaccine quality before it is administered to children and other beneficiary groups in the communities.

The cold chain is used for transporting and storing vaccines from manufacturers to vaccination points, using efficient cold chain systems and equipment to preserve vaccine quality [13, 14]. In addition, a strong immunisation supply chain is essential to improve coverage rates, and equity, and equally support strategies to reduce child mortality [13, 15, 16]. Healthcare systems worldwide rely on a competent supply chain system for the storage and transportation of health supplies at the right time, to the right place and in the proper condition equitably to all children for the welfare of all communities against vaccine-preventable diseases [17]. To ensure the effectiveness of vaccine supply, the health supply chains require effective management systems to handle vaccines and prevent wastage through providing healthcare workers with the necessary training and devices for vaccine management at all storage points [18–20].

In the Expanded Program on Immunization (EPI), the cold chain system is used for storing, transporting, and distributing vaccines and other biologicals in recommended temperatures until they reach beneficiaries [13, 21]. However, health workers and supervisors tend to emphasise protecting vaccines from heat damage, thus creating a risk of exposure to freezing temperatures. This practice may cause freezing during storage and distribution, damaging vaccines.

The World Health Organization estimated that about 67% of vaccine deliveries were likely to be damaged due to various factors, including absence of quality management practices and handling policies, with cold chain failures in 5 countries accounting for the loss of 2.8 million doses in 2011 [11, 22]. Furthermore, the survey conducted by the National Health Service of the United Kingdom reported that vaccine wastages cost around £6.3 million in 2019, of which 50% of wasted doses could have been avoided through the improvement of the cold chain system [23]. Likewise, the World Health Organization reported that most developing countries incur vaccine wastage costs of more than $4 million and $6 million, for pentavalent and pneumococcus vaccines, respectively, due to challenges associated with vaccine management practices [24].

Temperature management during the storage of vaccines is crucial to avoid wastage and preserve potency [25]. A study conducted in Nigeria reported that 75% of vaccines stored at subnational stores had sub-optimal quality and never improved, during three consecutive years of the study [24]. The findings were similar to the study conducted in Cameroon which reported that heat exposure during storage of vaccines at health facilities was higher compared to that observed at the district level, with the main contributors being the use of outdated and non-certified refrigerators, gaps in knowledge among healthcare workers, and their vaccine management practices [11, 26].

Although, the Tanzanian Effective Vaccine Management Assessment conducted in 2015 reported that Tanzania achieved an overall performance of 91% in temperature monitoring at the health facility level, there were some facilities that achieved less than a 60% score, which is below the minimum performance standard of 80% and above, for all criteria as recommended by WHO [27]. In addition, the Tanzania National Immunization Strategy (2021–2025) reported that temperature monitoring was not being performed effectively in terms of an inadequate number of devices, temperature charts not properly filled, and non-response to temperature alarms. However, the magnitude of challenges reported by the Ministry was not known [28]. We, therefore, conducted this study to assess vaccine management practices during the storage of routine childhood vaccines among healthcare workers at selected health facilities in the Morogoro region. The findings of this study may facilitate the improvement of healthcare workers’ knowledge and practices on vaccine management, thus improve the effective vaccine management assessment scores.

## Methods

### Study area

This study was conducted in the Morogoro region, found in the Eastern part of Tanzania. The three districts of Malinyi, Morogoro, and Gairo were purposively selected to represent three levels of immunization coverage. The facilities in Morogoro are providing routine childhood vaccination daily, while facilities with a low target population provide vaccination two to five times weekly. In addition, most facilities provide BCG and MR vaccines once to twice weekly depending on the number of children that attend sessions. This study was conducted at public health facilities that manage routine vaccines and provide immunization services to children and other beneficiaries including teenage girls, and pregnant women in the sampled districts of Malinyi, Morogoro and Gairo.

### Study design and population and sampling

A descriptive cross-sectional study was conducted using a quantitative approach. Data were collected using structured questionnaires that were administered to vaccine handlers and vaccinators. Participants in this study were healthcare workers with experience in vaccination or vaccine handling for not less than 1 year, and working at sampled facilities. This study involved 77 out of 345 facilities in three districts of Morogoro, Gairo and Malinyi that were providing routine childhood vaccination services to children below the age of 24 months.

A stratified random sampling technique was used, where facilities were put into three categories of best, intermediate and low performing based on the third-dose of DTP vaccination. Later, simple random sampling was used to select facilities from each category in each district. In each facility, study respondents were randomly selected, where one vaccinator was selected for facilities that had only health worker, and for facilities that had two or more vaccinators, the lottery method was used, where a vaccinator who picked a tossed paper with number one was selected and requested to respond to the questionnaire.

### Data collection and measurement

Data were collected using stock ledger reports that were found at vaccination facilities. The immunization and vaccine management system (VIMS), an electronic logistics management information system (eLMIS) managed by District Immunization and Vaccine Officers (DIVO), was used whenever there was missing stock data at the respective health facilities. In addition, temperature data were obtained from Fridge-tag® 2, 30DTR used for monitoring temperature devices using a structured observation form.

### Data analysis

Data collected during this study were entered into IBM Statistical Package for Social Studies (SPSS) and variables of interest including health workers training on vaccine handling, and cold chain management; functionality of the vaccine storage refrigerator; correct reading of the storage temperature from the temperature monitoring device, and reactions of health workers when they observe a freezing alarm in the temperature monitoring device, were analysed.

Descriptive analysis was performed for all demographic, and health facilities variables, as well as for study variables including health workers’ knowledge and practices on the vaccine and cold chain management, presence of refrigerators, characteristics and their performance, including the availability of an alternative source of energy.

## Results

### Demographic characteristics of participants

Findings from this study showed that the majority of participants 54 (70.1%) were females, compared to males 23 (29.9%) counterparts, with an education level of diploma, and certificate at 36 (46.8%), and 41 (53.2%), respectively. Other demographic variables are indicated in Table 1.

**Table 1:** Demographic characteristics of respondents

Variables	Quantities	Percent (%)
*Gender*		
Males	23	29.9
Females	54	70.1
*Level of education*		
Certificate	41	53.2
Diploma	36	46.8
*Profession*		
Nurse assistants	22	28.6
Nurse officers	31	40.3
Midwives	8	10.4
Other professions	16	20.8
*Experience in vaccination/vaccine management*		
Less than 5 years	30	39.0
5 years and more	47	61.0

### Characteristics of health facilities included in the study

It was observed that 65 (84%) refrigerators were functional, with over half of them being domestic models 43 (56%), followed by HBC-80 models 26 (34%) from Haier Biomedical. The study also reported the main sources of power for vaccine refrigerators to be 26 (34%), 28 (36%), and 23 (30%), for mains electricity, solar-powered and liquefied petroleum gas (LPG), respectively (Table 2). The study further found that 27 (35%) facilities had a backup source of power for vaccine refrigerators to ensure continuous storage of vaccines even when the main source goes off, while 50 (65%) had no backup.

**Table 2:** CCE models, functionality, alternative and main source of power for vaccine storage refrigerators

Variables	Quantities	Percent (%)
*Models of vaccine refrigerators*		
Vestfrost	1	1.0
Haier Biomedical (HBC-80 models)	26	34.0
Electrolux	3	4.0
Domestic	43	56.0
Unknown	4	5.0
*CCE functionality*		
Yes	65	84.0
No	12	12.0
*Source of power for vaccine refrigerators*		
Grid electricity	26	34.0
Solar power	28	36.0
Liquefied petroleum gas (LPG)	23	30.0
*Alternative power availability*		
Yes	27	35.0
No	50	65.0

### Storage temperature of vaccines at health facilities

**Fig. 1:**
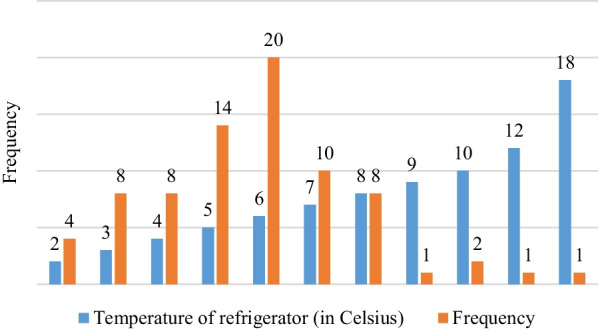
Proportion of refrigerators with their temperature records during the visit day (*n* = 77)

Data collected during this study showed that 72 (93.5%) facilities had their refrigerators working within WHO recommended temperature range of + 2° Celsius to + 8° Celsius (5.8 ± 2.431, Mean ± SE) (Fig. 1). The study further reported that there was no health facility that recorded temperature below + 2° Celsius, whereas 5 (6.5%) health facilities reported 9° Celsius to 18° Celsius temperature records until the day of data collection. Figure 1 provides more details.

Furthermore, the temperature records downloaded from Fridge-tag® 2 of health facilities that reported temperature exposure beyond + 8º Celsius found that health facilities 1 and 4 recorded the highest and the lowest cumulative exposure time of 281, and 5.9 h, respectively. It was further observed that these 5 health facilities reported 19 high-temperature alarms during the same period of data collection but none of them was recorded in the health facility’s temperature monitoring charts. Other facilities had exposure duration as indicated in Table 3.

**Table 3:** Temperature exposure of vaccine during storage at health facilities

Health facility	Duration (hours)
Facility 5	170.3
Facility 4	5.9
Facility 3	10.9
Facility 2	63.9
Facility 1	281.0

### Vaccine management practices by healthcare workers

#### Vaccine stock availability at health facilities

This study also assessed vaccine availability at health facilities for Bacille Calmette Guerin (BCG), Measles–Rubella (MR), Diphtheria–Tetanus–Pertussis–HepB–Hib (DTP–HepB–Hib), and Rotavirus for children, and Sinopharm for adults’ vaccination. Findings showed that most childhood vaccines were available in different proportions based on levels of consumption of health facilities (Table 4).

**Table 4:** Types and quantities of vaccine doses available at health facilities

	BCG	Rotavirus	DTP–HepB–Hib	MR	Sinopharm
*N*	77	77	77	77	77
Mean	95.9	46.2	73.8	62.3	47.9
Median	60	40	70	60	36
Mode	60	0^a^	70	0	0
Std. deviation	113	50.477	88.547	54.5	48.611
Minimum	0	0	0	0	0
Maximum	660	200	730	300	250

Multiple modes exist. The smallest value is shown

In addition, this study reported the availability of the COVID-19 vaccine, a non-childhood vaccine and Oxytocin a non-vaccine commodity, in 47 (61%), and 29 (36.6%) facilities, respectively. Furthermore, vaccine stockout was found in 12 (15.6%) health facilities for at least one antigen and 4 (5.1%) health facilities for all five antigens under observation. In general, all vaccines involved in this study experienced a stock-out status at one point in time during the data collection period (Table 5).

**Table 5:** Proportion of vaccine stock out at health facilities (*N* = 77)

Variables	Number of stocked facilities	Percent (%)
*Types of vaccines/commodities*		
BCG	10	13
PCV-13	10	13
Rotavirus	12	16
DTP–HepB–Hib	10	13
IPV	15	20
OPV	10	13
Td	11	14
MR	11	14
HPV	12	20
COVID-19	10	13
Hepatitis B	72	94
Oxytocin	29	38

#### Wastage of routine childhood vaccines at health facilities

This study monitored opened vial wastage of three routine childhood vaccines including DTP–HepB–Hib, Measles–Rubella (MR) and Rotavirus, with the wastage rate calculated using a formula developed by the WHO [10]. It was found that the average calculated vaccine wastage rates for DTP, Measles–Rubella and Rotavirus vaccines were 7%, 19%, and 15%, respectively, for the period of 6 month duration from March–August 2021 as indicated in Table 6.

**Table 6:** Calculated average wastage rates of DTP, Measles–Rubella and Rota vaccines

No	Name of district	Opened DTP doses	Number of children vaccinated	Calculated facility wastage rate (%)	Opened MR doses	Number of children vaccinated	Calculated facility wastage rate (%)	Opened Rota doses	Number of children vaccinated	Calculated facility wastage rate (%)
1	Malinyi district	12,530	11,584	7.5	7,770	6,311	18.8	8,139	6,964	14.4
2	Morogoro district	17,920	16,774	6.4	13,000	10,515	19.1	11,870	10,133	14.6
3	Gairo district	17,320	15,859	8.4	11,320	9,091	19.7	11,258	9,513	15.5
	Average vaccine wastage rate in the study area	47,770	44,217	7%	32,090	25,917	19%	31,267	26,610	15%

### Health workers’ knowledge during temperature monitoring

This study found that the majority of vaccinators 70 (91%) correctly mentioned the WHO-recommended storage temperatures of routine childhood vaccines at health facilities. However, there were 7 (9%) vaccinators who did not mention the correct recommended temperature range as indicated in Table 7. Health workers’ capacity to use electronic temperature devices during vaccine storage and take necessary actions needed to minimize wastage was also assessed. The results show that 58 (75%) health workers correctly knew how to read and record the current temperature of vaccine refrigerators from the Fridge-tag® 2 device. Likewise, it was reported that 55 (71%) and 51 (66%) health workers correctly read the maximum and minimum storage temperature of vaccines at refrigerators. Furthermore, the results indicated that lesser than half 38 (49%) of healthcare workers were reviewing the temperature records of their cold chain equipment (CCE) compared to 39 (51%) who did not review their temperature records as shown in Table 7. In addition, the majority of vaccinators, 63 (82%) mentioned safety and quality monitoring as the key functions of VVM sticker, while collectively 14 (18%) vaccinators mentioned other functions. Concerning vaccines that are easily affected by freezing temperature, the first, second and third most reported vaccines were TT/Td, PCV-13 valent, and DTP–HepB–Hib with 30%, 44%, and 74%, respectively.

**Table 7:** Knowledge of HCWs on functions of a VVM indicator, and freeze-sensitive vaccines; and reading a Fridge-tag® 2 device

Variables	Number	Percent (%)
*Functions of vaccine vial monitors (VVM)*	*n* = 77	
Prioritize vaccine vials for usability	2	3.0
Monitor refrigerator performance	11	14.0
Assess vaccine safety	17	22.0
Assess vaccine quality during storage	46	60.0
Assess heat exposure to vaccine	1	1.0
*VVM sticker found at/or beyond the discard point*	*n* = 77	
Yes	10	13.0
No	67	87.0
*Know the WHO recommended storage temperatures of vaccine (+ 2 to + 8ºC)*	(*n* = *77*)	
Yes	70	90.9
No	7	9.1
*Knowledge on temperature monitoring*		
Reading current temperature from a Fridge-tag® 2	*n* = 77	
Yes	58	75.0
No	19	25.0
Reading minimum temperature from a Fridge-tag® 2	*n* = 77	
Yes	51	66.0
No	26	34.0
Reading maximum temperature from a Fridge-tag® 2	*n* = 77	
Yes	55	71.4
No	22	28.6
*Health workers’ performing monthly temperature data reviews*		
Yes	38	49.0
No	39	51.0
*Knowledge of freeze-sensitive vaccines in a refrigerator*		
TT/Td	23 (*n* = *77*)	30.0
PCV	34 (*n* = *77*)	44.0
DTP	57 (*n* = *77*)	74.0

## Discussion

This study aimed to assess vaccine management practices among healthcare workers at health facilities in the Morogoro region.

The findings revealed that most facilities had a well-functional refrigerator that worked within WHO recommended temperature range of + 2 to + 8 °C, with the source of power being solar, grid and LPG at almost equal proportions. The findings of this study were higher than a study conducted in Ethiopia [9] that reported 30–50% of the refrigerators and freezers being out of order. However, the findings of this study were observed to be slightly lower compared to a similar survey conducted in Nigeria [29]. Available cold chain equipment (CCE) is essential for effective vaccine management to avoid wasting thermo-sensitive products such as vaccines during storage. The presence of non-functional CCE also may indicate a lack of planned preventive maintenance and immediate findings and increase unnecessary travel frequencies to facilities for children to be fully vaccinated.

To ensure operating vaccine CCE within the recommended temperatures, the facilities must have a reliable source of power supply as well as a backup power source to ensure service continuity. In this study, the rate of alternative power availability was half that of a similar survey conducted in the Nigeria [29]. In contrast, it was slightly lower than that undertaken in the Ethiopia [9]. The absence of an alternative source of power for refrigerators might be due to a lack of policy to ensure effective vaccine management at health facilities. If not well-addressed, this observation might cause wastage of vaccines, increase operational costs to facilities, because vaccines have to be moved to nearby facilities with power and sometimes delay children from being fully and timely vaccinated against vaccine-preventable diseases.

Moreover, the proportion of facilities with vaccine vials with VVM stickers at the discard point or beyond was lower than that reported by similar studies conducted in Nigeria and Ethiopia [9, 29, 30]. Furthermore, the presence of vials with VVM beyond the discard point in this study indicates poor cold chain management and is contrary to the findings of a study done in Nigeria [29]. Although this result is encouraging, it shows inadequate knowledge among healthcare workers. This might be due to the lack of training for vaccinators in Tanzania, particularly in the Morogoro region. This observation might be linked to the common knowledge of healthcare workers in interpreting the colour change of VVM stickers during vaccine storage, increasing vaccine wastage and delaying the national goal to vaccinate all children in all communities and avoid missed opportunities for vaccination (MOV) [31].

Various studies in Africa and other parts of the world identified weaknesses in cold chain management that influence vaccine wastages, including shared knowledge among health providers, breakdown of the cold chain systems, power interruption, and quality of refrigerators [9, 32–34]. In this study, the vaccine wastage level was lower than those reported in other studies conducted in India, which reported a wastage of DTP and MR to be 32.1% and 32.2%, and 25.4%, and 21.7%, for Kangra and Pune districts, respectively [35]. However, the observed wastage level was beyond the national guidelines, implying the need for health workers’ training on strategies to minimise wastage at facilities during storage and service delivery. It is assumed that target children for immunisation are missing immunisation services and hence not protected as some vaccinating facilities miss any antigen of interest.

Adequate knowledge and skills in temperature monitoring are essential for healthcare workers to review health facility temperature data, establish response actions to avoid and minimise vaccine wastages during storage, and build community confidence in immunisation services. It was found that health workers have limited knowledge of proper cold chain management, with shallow scores in identifying vaccines that are mostly affected by freezing temperatures, compared to the study conducted in Ethiopia [21] and Nigeria [32]. The lack of knowledge might be attributed to a lack of health workers training on cold chain management to enable effective performance according to guidelines.

The vaccine management practices at health facilities were reported to be inefficient due to issues including inadequate knowledge and skills of healthcare workers, as was observed through failure to read temperature monitoring devices, lousy recording of temperature records and follow-up of minimum and maximum temperature records. Storage temperature data reported by this study indicated weaknesses in the vaccine storage at health facilities causing exposure to high temperatures that caused vaccine wastage after the change of VVM colour beyond the discard point.

Since this study did not report exposure of vaccines to freezing temperatures, we recommend a longitudinal study using continuous temperature monitoring devices and vaccine wastage assessment to identify the magnitude of the problem and the contributing factors in both closed and opened vials to guide management and policy decisions.

To maintain effective cold chain management, further administrative efforts, including training healthcare providers, are compulsory to improve the knowledge and skills reported by this study. District Health Management authorities should ensure that necessary steps are made to ensure vaccine wastage data are routinely captured and evaluated at each facility using data quality self-assessment (DQSA). Besides, health facilities should establish and monitor data on vaccine wastage because of its importance in vaccine forecasting, decision-making toward introducing new and more expensive vaccines and ensuring stock availability in all communities.

Some facilities in the study area experienced vaccine stock out for at least one antigen, and all five antigens are under observation. Given the recommendation per the national vaccination policy to ensure access and utilisation of immunisation services, every facility was requested to maintain a 50% or 2-week buffer stock of vaccines at all times. The reported stockouts of vaccines would indicate that safety stock levels have been depleted and that vaccine availability could be compromised and lead to outbreaks of vaccine-preventable diseases.

In addition, the researchers calculated the average vaccine wastage rates for DTP–HepB–Hib, Measles–Rubella vaccine (MR) and Rotavirus (RV) vaccines. Healthcare providers must monitor vaccine wastages and document various reasons so interventions can be instituted to minimise observed levels. The national immunisation policy mandates monthly monitoring of vaccine wastages at all levels and establishes strategies to reduce wastages without compromising the vaccination of clients in all communities. The findings of this study further indicated that wastage of vaccines in unopened vials of DTP–HepB–Hib, and Measles–Rubella (MR) vaccines were within national limits and thus may be considered acceptable.

### Limitations of the study and future research

First, there was limited access to stock data from some healthcare facilities due to inconsistencies in availability and regular updating of tools from primary data sources, the vaccine stock ledgers. However, the researchers decided to incorporate the electronic vaccination and vaccine stock management system available at the district offices, which delivered vaccine stocks and immunization-related supplies to health facilities. Second, this study was conducted in only 77 facilities. Thus, there was a reduced statistical power. Therefore, caution is needed when generalising these findings to all health facilities. Future research needs to be conducted to replicate these findings using all health facilities. In addition, this study did not go further to identify reasons for observed vaccine stockouts or specific reasons for observed wastage at health facilities. Therefore, future studies that explore these factors are highly encouraged.

## Conclusions

This study aimed to assess vaccine management practices among healthcare workers at health facilities in the Morogoro region. The findings indicated that most health facilities have a well-functional refrigerator and use power. However, less than half of the health facilities have a backup plan. Healthcare workers’ knowledge of the vaccine and cold chain management was quite good for temperature data reading and documentation. However, the practices were poor for some health facilities. The gaps observed in this study indicate a need to improve the knowledge and practice of health workers, including mentorships, supervisions, and training to guarantee that vaccines are well kept and maintain potency, so that every child in all communities reaps the benefits of immunization services.

## Data Availability

All the data used for this study are available upon a reasonable request from the first author.

## Abbreviations

eLMIS: Electronic Logistics Management Immunization Systems
EPI: The Expanded Program on Immunization
EVMA: Effective Vaccines Management Assessment
CCE: Cold Chain Equipment
CHMTs: Council Health Management Teams
DIVO: District Immunization and Vaccines Officer
DTP-3: The 3-dose of diphtheria, tetanus and pertussis vaccines
30DTR: 30-Day Temperature Recorder
RHMT: Region Health Management Team
RTMDs: Remote Temperature Monitoring Devices
VIMS: Vaccine Information and Management System
WHO: The World Health Organisation
